# Predicting corn digestible and metabolizable energy content from its chemical composition in growing pigs

**DOI:** 10.1186/2049-1891-5-11

**Published:** 2014-02-13

**Authors:** Quanfeng Li, Jianjun Zang, Dewen Liu, Xiangshu Piao, Changhua Lai, Defa Li

**Affiliations:** 1 State Key Laboratory of Animal Nutrition, Ministry of Agriculture Feed Industry Centre, China Agricultural University, Beijing 100193, China

**Keywords:** Corn, Digestible energy, Metabolizable energy, Pigs, Prediction equation

## Abstract

**Background:**

The nutrient composition of corn is variable. To prevent unforeseen reductions in growth performance, grading and analytical methods are used to minimize nutrient variability between calculated and analyzed values. This experiment was carried out to define the sources of variation in the energy content of corn and to develop a practical method to accurately estimate the digestible energy (DE) and metabolisable energy (ME) content of individual corn samples for growing pigs. Twenty samples were taken from each of five provinces in China (Jilin, Hebei, Shandong, Liaoning, and Henan) to obtain a range of quality.

**Results:**

The DE and ME contents of the 100 corn samples were measured in 35.3 ± 1.92 kg growing pigs (six pigs per corn sample). Sixty corn samples were used to build the prediction model; the remaining forty samples were used to test the suitability of these models. The chemical composition of each corn sample was determined, and the results were used to establish prediction equations for DE or ME content from chemical characteristics. The mean DE and ME content of the 100 samples were 4,053 and 3,923 kcal/kg (dry matter basis), respectively. The physical characteristics were determined, as well, and the results indicated that the bulk weight and 1,000-kernel weight were not associated with energy content. The DE and ME values could be accurately predicted from chemical characteristics. The best fit equations were as follows: DE, kcal/kg of DM = 1062.68 + (49.72 × EE) + (0.54 × GE) + (9.11 × starch), with R^2^ = 0.62, residual standard deviation (RSD) = 48 kcal/kg, and *P* < 0.01; ME, kcal/kg of dry matter basis (DM) = 671.54 + (0.89 × DE) – (5.57 × NDF) – (191.39 × ash), with R^2^ = 0.87, RSD = 18 kcal/kg, and *P* < 0.01.

**Conclusion:**

This experiment confirms the large variation in the energy content of corn, describes the factors that influence this variation, and presents equations based on chemical measurements that may be used to predict the DE and ME content of individual corn samples.

## Background

Corn is the principal cereal grain used in swine diets because it is widely grown, have highly DE and ME, and is generally economical. However, variation in nutrient content of corn has the potential to greatly affect profits in pig production. For example, variation in valuable energy may translate to economically significant changes in feed conversion [1]. To prevent unforeseen reductions in growth performance, grading and analytical methods are used to minimize nutrient variability between calculated and analyzed values. In the present Chinese grading system, corn is graded based on bulk weight and damaged kernels even though other factors may affect its feeding value.

Nutrient digestibility of corn is affected by agronomic conditions, genetics, postharvest processing, storage conditions, and anti-nutritional factors [2,3]. Differences between corn samples can yield variability in available energy, nutrient digestibility and growth performance in pigs [4-6]. However, the United States department of agriculture (USDA) corn grading system and Chinese grading system are based primarily on physical characteristics, such as bulk weight. Therefore, corn is priced with disregard to variations in chemical quality due to the vast scale of analysis that would be required commercially [6] and the acceptance that nutrient value of feed ingredients may be constant based on broad-based quality designations [7].

By potentially ignoring inherent variation in nutrient content and digestibility, the grading methods used to evaluate corn, such as bulk weight, may be poor estimators of feeding value [8,9]. Furthermore, prediction equations for digestible energy (DE) and metabolisable energy (ME) in feed ingredients based on chemical composition can be a useful tool in feed ingredient evaluation, but such equations are currently available only for barley [3], DDGS [10,11], wheat [12], and complete diets [13]. To our knowledge, there is a lack of peer-reviewed information regarding the combination of these techniques to predict nutrient digestibility of diverse samples of corn in pigs. The objectives of the present study were to characterize the nature of the variation in the energy content of corn and to develop a system(s) that accurately estimates the DE and ME levels in individual corn samples.

## Methods

### Selection and preparation of the corn samples

The corn samples were obtained from the main corn producing areas of China. Jilin, Liaoning, and northern Hebei provinces are spring corn-growing areas; seeds are planted from the end of April to May. Southern Hebei, Shandong and Henan provinces are summer corn-growing areas; seeds are planted in mid-June. To obtain a range of quality of Chinese feed corn, a total of 100 corn samples were taken, twenty from each of five provinces (Jilin, Hebei, Shandong, Liaoning, and Henan). From each location, one sample was selected to be below average and another was selected to be above average in bulk weight. Thus, the primary goal of the sample selection process was not to compare cultivars, but rather to provide a diverse array of corn samples for investigation into the nature of energy variability in corn. The chemical characteristics and physical characteristics of corn are shown in Table 1. The Institutional Animal Care and Use Committee at China Agricultural University (Beijing, China) reviewed and approved the protocols used in this study.

**Table 1:** Chemical and physical characteristic characteristics of 100 corn samples

**Item**	**Mean**	SD^*^	**CV**^**※**^	**Minimum**	**Maximum**
Chemical composition, % of DM					
Crude protein	9.69	0.50	5.16	7.78	11.03
Ash	1.36	0.14	10.07	0.99	1.79
Calcium	0.03	0.01	26.67	0.01	0.03
Phosphorus	0.25	0.03	11.20	0.18	0.32
NDF	11.13	0.93	8.35	9.56	17.36
ADF	2.29	0.23	10.04	1.86	2.95
Ether extract	3.65	0.54	14.79	2.04	4.81
Starch	72.77	3.32	4.56	53.46	79.80
Gross energy, kcal/kg of DM	4,447	44.96	1.01	4,357	4,537
Physical characteristic					
Bulk weight, g/L	698.68	25.91	3.71	573.62	752.35
1,000 kernel weight, g	325.48	41.16	12.64	220.20	411.10

^*^SD, Standard deviation; ^※^CV, Coefficient of variation.

### Experimental design

Sixty corn samples were randomly selected from the five provinces of China, every province contains twelve samples, to develop a prediction model for DE and ME that could be utilized for the formulation of diets for pigs. The remaining forty corn samples were used to test the accuracy of the DE prediction model. One hundred diets were formulated to contain 96.8% of one of each of the corn samples and 3.2% minerals and vitamins (Table 2). Corn was assumed to be the only source of energy in the diet as the slight contribution of energy from vitamin and mineral premixes was assumed to be negligible. Vitamins and minerals were supplied at levels formulated to exceed the requirements of 20 to 50 kg growing pigs as defined by NRC [14].

**Table 2:** Composition of the experimental diets (as-fed basis) fed to growing pigs for comparison of the energy digestibility between different corn samples

**Ingredient**	**%**
Corn	96.8
Antioxidant^1^	0.1
Dicalcium phosphate	1.7
Limestone	0.6
Salt	0.3
Vitamin and trace mineral premix^2^	0.5

^1^Santoquin MAX composite antioxidant, contained no less than 10% Ethoxyquin, no less than 3% Butylated Hydroxytoluene (BHT) and Citric acid, provided by Novus International, Inc.

^2^Premix provided the following per kg of complete diet for growing pigs: vitamin A, 5,512 IU; vitamin D_3_, 2,200 IU; vitamin E, 30 IU; vitamin K_3_, 2.2 mg; vitamin B_12_, 27.6 μg; riboflavin, 4 mg; pantothenic acid, 14 mg; niacin, 30 mg; choline chloride, 400 mg; folacin, 0.7 mg; thiamine 1.5 mg; pyridoxine 3 mg; biotin, 44 μg; Mn, 40 mg (MnO); Fe, 75 mg (FeSO_4_ · H_2_O); Zn, 75 mg (ZnO); Cu, 100 mg (CuSO_4_ · 5H_2_O); I, 0.3 mg (KI); Se, 0.3 mg (Na_2_SeO_3_).

The total experiment consisted of five digestibility trials conducted from October 2011 to March 2012 under similar experimental conditions. We have ten metabolism rooms, and each room has twelve metabolism cages. Six workers were employed to collect feces. Each successive trial measured twenty diets. A total of six hundred crossbred barrows (Duroc × Landrace × Yorkshire) (initial BW, 35.3 ± 1.9 kg) were used according to a completely randomized design, and each diet was tested with six pigs. Pigs were individually housed in stainless steel metabolism cages (1.4 m × 0.45 m × 0.6 m), and were weighed at the beginning of each period. Pigs were adapted to the diet and the digestibility cage for more than ten days before total collection of feces and urine for five days. The crates were located in an environmentally controlled room with a temperature of 22 ± 1°C.

Feed was provided twice daily at 08:00 and 17:00 h as a mash. Water was continuously available through a nipple drinker. During a ten days period of adjustment to the metabolism crates and diets, average daily feed intake was gradually increased until it was estimated to supply 4% of the average BW determined at the initiation of each adaptation period. Feed refusals and spillage were collected daily and weighed. The collection and sample preparation of feces and urine were conducted according to the methods described by Song et al. [15]. Feces were collected as they appeared in the metabolism crates and placed in plastic bags to be stored at −20°C. Urine was collected in a bucket placed under the metabolic crate. The bucket contained 10 mL of 6 mol/L HCl for every 1,000 mL of urine. Each day, the total urine volume was measured and a 10% aliquot was filtered through gauze and the urine samples were transferred into a screw-capped tube and immediately stored at −20°C until needed for analysis. At the end of the collection period, feces were thawed, pooled by pig within period, homogenized, sub-sampled, dried for 72 h in a 65°C drying oven and ground through a 1-mm screen. For analysis, all the corn samples were ground through a 1-mm screen as well.

### Chemical analyses

All chemical analysis were conducted in duplicate and repeated if the results differed by more than 5%. The ingredients used in this experiment were analyzed for dry matter (DM) [16], ether extract (EE) [17], ash [16], calcium [16], and phosphorus [16]. Kjeldahl N was determined according to the method used by Thiex et al. [18]. The content of neutral detergent fibre (NDF) and acid detergent fibre (ADF) were determined using filter bags and fiber analyzer equipment (Fiber Analyzer, Ankom Technology, Macedon, NY) following a modification of the procedure of Van Soest et al. [19]. Starch content was determined after converting starch to glucose using an enzyme assay kit (Megazym International Ireland, Wicklow, Ireland). The GE of feces, diets and corn samples were measured using an automatic adiabatic oxygen bomb calorimeter (Parr 6300 Calorimeter, Moline, IL). The GE of urine was measured by injecting 4 ml of the sample into 2 filter papers in a special crucible, and dried for 8 h in a 65°C drying oven to determine the energy. The 1,000-kernel weight (g/1,000 seeds) was measured in each sample of test corn by first cleaning it of all foreign materials and then counting 1,000 seeds.

### Calculations and statistical analysis

The apparent total tract digestibility (ATTD) of GE was measured on the 100 feed samples and was later converted to reflect the digestibility of the individual corn sample. The small portion of the experimental diets that consisted of minerals and vitamins (3.2%) was assumed to have a negligible contribution to the digestibility of GE.

This experiment was a completely randomized design; the data were analyzed using the mean, correlation, GLM, and one-way ANOVA procedures of SAS (SAS Inst. Inc., NC). The individual animal and corn sample were the experimental units for analyzing the data from the digestibility trial and analysis of the chemical constituents, respectively. The relationship between physical characteristic, chemical composition, DE and ME were analyzed using the CORR procedures of SAS (1991). The linear regression equations for predicting the DE and ME value of the corn from the chemical constituents were calculated with the forward stepwise regression procedure within SAS (1991). The level of significance adopted was 5% (*P* < 0.05). The equations with the smallest RSD are presented in the results.

## Results

### Chemical characteristics, physical characteristics and gross energy of corn

As expected, the chemical composition and physical characteristics of corn were quite variable for some criteria (Table 1). On a dry matter basis, the concentration of CP ranged from 7.78 to 11.03% with a mean of 9.69%. Ash concentration ranged from 0.99 to 1.79% with a mean of 1.36%. The variation was particularly high within the main fiber fractions as NDF concentration in corn ranged from 9.56 to 17.36% (mean 11.13%) of DM, while values for ADF in corn ranged from 1.86 to 2.95% (mean 2.29%) of DM. Concentrations of EE and starch varied greatly as well, ranging from 2.04 to 4.81% and from 53.46 to 79.80% and averaging 3.65 and 72.77%, respectively. In contrast, GE content of the corn samples varied slightly. The bulk weight of corn ranged from 573.62 to 752.39 g/L (mean 698.68 g/L). The 1,000-kernel weight varied greatly as well, ranging from 220.20 to 411.10 g (mean 325.48 g).

### Energy concentration and energy digestibility of corn

Energy concentration and the ATTD of GE of the corn are shown in Table 3. In the 100 corn samples, DE content ranged from 3,931 to 4,180 kcal/kg with a mean DE content of 4,053 kcal/kg, resulting in a 6% range in DE. The ME content ranged from 3,798 to 4,092 kcal/kg with a mean ME content of 3,923 kcal/kg, and the overall variation in ME was 294 kcal. The ratio of ME to DE calculated from 100 measured samples ranged from 95.41 to 98.13% with a mean value of 96.78%. The ATTD of GE ranged from 83.43 to 92.25 % with a mean of 90.49%.

**Table 3:** Energy concentration and ATTD of GE of the 100 corn samples

**Item**	**Mean**	**Minimum**	**Maximum**	**SD**	**CV**
DE, kcal/kg of DM	4,053	3,931	4,180	60.39	1.49
ME, kcal/kg of DM	3,923	3,798	4,092	55.42	1.41
ME/DE	96.78	95.41	98.13	1.01	1.04
ATTD of GE, %	91.15	83.27	93.07	0.97	1.06

### Effect of growing region on chemical characteristics, physical characteristics and energy values

With exception to ash content and bulk weight (Table 4), the chemical characteristics, physical characteristics and energy values of corn were influenced significantly by growing region (*P* < 0 . 01). Among five provinces, Henan had the highest starch, NDF and energy content (GE, DE and ME) and corn had a larger 1,000-kernel weight when grown in the Liaoning (354.59 g) and Jilin (363.60 g) provinces compared with corn grown in the Henan (299.70 g) and Shandong (307.38 g) provinces. The DE content of corn grown in Liaoning (4,032.69 kcal/kg) and Jilin (4,035.85 kcal/kg) provinces were similar; however, the DE content of corn grown in Shandong (3,996.61 kcal/kg) province was lowest. Overall, growing region significantly influenced the DE content of corn (*P* < 0 . 01). Corns grown in the spring growing areas (Liaoning and Jilin provinces) had a significantly higher 1,000-kernel weight compared with corn grown in the summer growing areas (Henan and Shandong provinces).

**Table 4:** Effect of growing region on chemical characteristics, physical characteristics and energy values of the 100 corn samples from five provinces

**Item**	**Liaoning**	**Jilin**	**Hebei**	**Henan**	**Shandong**	**SEM**	***P*-value**
Chemical composition, % of DM							
Dry matter	87.57^b^	88.15^a^	86.82^c^	86.49^d^	88.20^a^	0.47	0.01
Crude protein	9.87^a^	9.78^a^	9.38^b^	9.61^ab^	9.82^a^	0.47	0.01
Ether extract	3.54^b^	3.38^b^	4.05^a^	3.71^b^	3.56^b^	0.05	0.01
ADF	2.28^b^	2.22^b^	2.46^a^	2.29^b^	2.25^b^	0.22	0.01
NDF	10.95^bc^	10.73^c^	10.76^c^	11.76^a^	11.46^ab^	0.86	0.01
Ash	1.61	1.62	1.58	1.60	1.63	0.10	0.55
Starch	70.58^c^	73.20^b^	72.97^b^	75.32^a^	71.79^bc^	2.97	0.01
Physical characteristic							
Bulk weight, g/L	693.29	689.94	699.21	707.94	703.04	25.60	0.18
1,000 kernel weight, g	354.69^a^	363.60^a^	302.19^b^	299.70^b^	307.38^b^	30.92	0.01
Energy concentration, kcal/kg of DM							
Gross energy	4448.16^b^	4436.57^b^	4435.73^b^	4476.82^a^	4436.69^b^	18.23	0.01
Digestible energy	4032.85^b^	4035.85^b^	4093.45^a^	4106.23^a^	3996.61^c^	45.20	0.01
Metabolisable energy	3906.83^b^	3903.14^b^	3970.06^a^	3968.63^a^	3868.94^c^	40.61	0.01

a-d Means followed by the same letter within each row are not significantly different from each other (*P* > 0.05).

### Correlation coefficients between physical and chemical characteristics and energy values

In the 100 corn samples, fibrous compounds had a negative correlation with DE and ME content, while the correlation of EE, GE and starch with DE content was positive (Table 4). The content of EE had the highest correlation of any characteristic with DE content (r = 0.44; *P* < 0.01), followed by total starch (r = 0.38; *P* < 0.01), NDF (r = −0.32; *P* < 0.01) and ash (r = −0.29; *P* < 0.05). Correlation analyses showed that ME content of corn was positively correlated to the DE (r = 0.95; *P* < 0.01) and EE content (r = 0.29; *P* < 0.01), while ash (r = −0.28, *P* < 0.01) and NDF (r = −0.27, *P* < 0.01) had a negative correlation with ME content. The correlation of bulk weight and 1,000-kernel weight with energy content was not significant (Table 5).

**Table 5:** Correlation coefficients between chemical characteristics, physical characteristics and energy values of the 100 corn samples

**Item**	**DE**	**ME**	**Crude protein**	**Ether extract**	**ADF**	**NDF**	**Ash**	**Gross energy**	**Starch**	**Bulk weight**	**1,000 kernel weight**
DE	1.00										
ME	0.95**	1.00									
Crude protein	−0.15	−0.06	1.00								
Ether extract	0.44**	0.29**	−0.28	1.00							
ADF	−0.05	−0.06	−0.05	0.33	1.00						
NDF	−0.32**	−0.27**	0.14	−0.02	0.68	1.00					
Ash	−0.29*	−0.28**	0.22	−0.27	−0.11	0.17	1.00				
Gross energy	0.25**	0.23	−0.06	0.14	−0.28	0.19	0.07	1.00			
Starch	0.38**	0.32*	−0.03	0.03	0.13	0.11	−0.12	0.06	1.00		
Bulk weight	0.05	0.03	0.03	0.15	−0.16	0.13	0.22	0.05	−0.13	1.00	
1,000 kernel weight	0.11	0.06	0.25	0.02	−0.06	−0.13	0.08	0.14	−0.11	0.42	1.00

*, **, *P* < 0.05, *P* < 0.01, respectively.

### Prediction equations for digestible energy and metabolizable energy

Some equations based on simple and multiple linear regression analysis were then conducted to develop prediction equations for DE content of corns based on the results of stepwise regression analysis (Table 6). According to the high correlation between DE and EE content (Table 5), the best single predictor was always the EE estimate. Prediction slightly improved when the starch content was included (Equation 2 in Table 6). Addition of NDF and GE content to the equation improved the precision of the prediction (Equations 3 and 4 in Table 6). Among the different predictors, the predictions with the lowest RSD were obtained when EE, starch, NDF, and GE were considered (Equation 4 in Table 6). The residual standard deviation (RSD) was then equivalent to 48 kcal of DM.

**Table 6:** Most effective prediction equations of digestible energy (kcal/kg; dry matter) based on chemical variables (% or kcal/kg; dry matter) of the corn samples

**No.**	**Equation**	**R**^**2**^	**RSD**	***P*-value**
1	DE = 3889.81 + (46.21 × Ether extract)	0.20	59	<0.01
2	DE = 3335.46 + (44.91 × Ether extract) + (7.59 × Starch)	0.33	58	<0.01
3	DE = 3507.46 + (44.01 × Ether extract) – (20.09 × NDF) + (8.38 × Starch)	0.45	56	<0.01
4	DE = 1062.68 + (49.72 × Ether extract) – (24.89 × NDF) + (0.54 × Gross energy) + (9.11 × Starch)	0.62	48	<0.01

Equations for estimating ME content from chemical characteristics were calculated similarly. The results showed that the ME content of corn could be predicted with a reasonable degree of accuracy by measuring the DE (Equation 9 in Table 7). As with DE, the addition of NDF and ash content to the equation improved the precision of the prediction (Equations 10 and 11 in Table 7). The content of ME can also be accurately predicted from CP, EE, NDF and ash contents without DE content (RSD, 35 kcal/kg of DM) (Equation 8 in Table 7).

**Table 7:** Most effective prediction equations of ME (kcal/kg; DM) based on chemical variables (% or kcal/kg; DM) of the corn samples

**No.**	**Equation**	**R**^**2**^	**RSD**	***P*-value**
5	ME = 4190.19 – (19.56 × NDF)	0.22	44	<0.01
6	ME = 4664.04 – (17.74 × NDF) – (310.93 × Ash)	0.33	40	<0.01
7	ME = 4464.24 + (20.15 × Ether extract) – (17.84 × NDF) – (233.72 × Ash)	0.44	39	<0.01
8	ME = 4289.74 + (20.02 × Crude protein) + (22.47 × EE) – (18.40 × NDF) – (245.20 × Ash)	0.49	35	<0.01
9	ME = −213.69 + (1.02 × DE)	0.79	23	<0.01
10	ME = 362.67 + (0.95 × DE) – (193.59 × Ash)	0.85	19	<0.01
11	ME = 671.58 + (0.89 × DE) – (5.57 × NDF) – (191.39 × Ash)	0.87	18	<0.01

### Comparison of DE content in corn determined by using the *in vivo* method and prediction model

To test the suitability of these models (Table 6) to predict the DE content of a normal corn sample, the DE content of 40 samples of corn was measured by both the *in vivo* method and prediction models (Equation 4 in Table 6). Our results showed that the maximum absolute difference between DE determined by the *in vivo* method and the prediction model was 104.61 kcal/kg, while the minimum absolute difference was 0.15 kcal/kg (Table 8). The mean of observed group and prediction group were 4,035.05 and 4,021.73 kcal/kg, and the difference was only 13.32 kcal/kg. Therefore, the prediction models established from 60 corn samples as described in this article can be used to predict the DE content of corn for pigs with acceptable accuracy.

**Table 8:** Comparison of digestible energy contents in corn determined by using the *in vivo* method and prediction model (kcal/kg of DM)

**Test**	**Observed**	**Predicted**	**Difference**
1	4,084.05	3,996.40	87.64
2	4,108.22	4,040.57	67.65
3	4,140.16	4,101.24	38.92
4	4,023.51	4,019.85	3.66
5	4,113.16	4,047.87	65.29
6	3,950.07	4,050.91	−100.84
7	4,058.73	4,085.59	−26.85
8	4,054.97	4,062.27	−7.30
9	4,046.38	4,007.67	38.71
10	3,997.07	3,989.82	7.25
11	4,044.88	3,969.98	74.89
12	4,062.20	4,042.23	19.97
13	4,062.67	3,994.71	67.95
14	4,009.57	3,974.33	35.23
15	3,957.41	3,962.92	−5.51
16	4,036.46	4,036.31	0.15
17	3,983.54	3,970.58	12.96
18	4,011.42	3,993.93	17.49
19	3,932.03	3,951.91	−19.88
20	3,980.49	4,035.48	−54.99
21	4,065.07	4,054.47	61.22
22	4,024.33	4043.60	−19.28
23	4,025.92	4,020.54	5.38
24	4,020.02	4,024.04	−4.02
25	4,016.88	4,005.85	11.03
26	4,051.08	3,996.01	55.06
27	4,058.55	3,987.99	70.57
28	4,051.18	4,018.28	32.90
29	4,088.96	4,036.06	52.90
30	4,087.01	4,028.30	58.71
31	4,121.96	4,017.35	104.61
32	4,084.62	4,055.96	28.66
33	4,002.90	4,050.52	−47.62
34	3,979.24	4,014.76	−35.52
35	4,003.71	4,022.21	−18.50
36	4,066.90	4,022.56	44.34
37	3,934.52	4,003.62	−69.10
38	4,041.55	4,038.95	2.59
39	4,029.50	4,062.03	−32.54
40	3,991.01	4,031.85	−40.84
SD^*^	49.74	32.93	
RSD^※*^			46.75
*P*-value			0.16

*SD, Standard deviation; ^※^RSD, residual standard deviation.

## Discussion

### Chemical characteristics, physical characteristics and energy variation in corn

The chemical composition and concomitant nutritional value of corn is variable and dependent on variety, growing environment, drying temperature, starch structure and the presence of various anti-nutritive factors [8,20-27]. Comparing the present study with the National Research Council [28], mean GE concentration was identical, mean CP and starch concentrations were higher than the NRC (2012) values, mean EE and ADF concentrations were lower than the NRC (2012) values. Content of GE did not vary much among the 100 samples. The CV for CP, NDF, starch, and GE were within 10%, but wide variations in the content of EE (CV: 14.79%), ash (CV: 10.07%), Ca (CV: 26.67%), P (CV: 11.20%) and ADF (CV: 10.04%) were observed (Table 1). For the physical trait, the wide variation in 1,000-kernel weight also was observed (CV: 12.64%).

Variety and growing environment are major sources of variation for the chemical composition of cereal grains [29]. Table 4 also indicated that growing regions influenced significantly the chemical characteristics, physical characteristics and energy values of corn. A previous study demonstrated that chemical composition, especially the NSP composition and structure, was significantly different due to variety and growing location and was negatively correlated with the DE content [30]. One reason for not observing a huge degree of variation in some criteria may be that no extreme samples were collected, such as high-oil corn, high-lysine corn, or NutriDense corn. The other reason is that the quality standards for corn were similar for all the feed companies who collected the samples for this study.

Corn samples were collected for use in this study to increase the likelihood of observing a degree of variation in DE and ME content of the 100 corn samples. Yellow Dent corn in the NRC [28] has a DE value of 3,921 kcal/kg of DM and a ME value of 3,857 kcal/kg of DM, respectively. The average DE for corn determined in the present study was 4,053 kcal/kg of DM, and ME averaged 3,923 kcal/kg of DM, respectively. The difference between the current study and previous results could be attributable to differences in particle size of corn. The mean particle size of the corn reported herein was 441 μm, well below the 600 to 700 μm level recommended for corn fed to growing swine [31,32]. Data reported by Owsley et al. [33], and Healy et al. [32] suggest that the DE of diet increases when particle size is reduced. The screen was a new one for this study, and Chinese producers prefer the small feed particles.

The amount of energy lost in urine represented from 2 to 5% of dietary DE content (mean: 3.0%). The mean ME:DE ratio therefore averaged 97%. The ME:DE ratio obtained in the current study corresponds with the data published by NRC [28], which reported Yellow Dent corn had an ME:DE ratio of 98%. Based upon the NRC [28] reported value, Noblet and Perez [13] proposed an equation (ME/DE = 100.3 - 0.21 × CP) to be applied to feed ingredients. Under practical conditions, the dietary CP content is less variable, so ME:DE has been considered almost as a constant of approximately 96.78% [34]. The mean of the ATTD of GE determined for these corn samples was 91.15%, somewhat higher than standard values, which are typically 87.74% (Yellow Dent Corn) [28]. One possible explanation for the higher ATTD of GE could be the smaller than expected particle size of the ground corn.

### Factors influencing energy variation

Corn is typically sold as a commodity and is valued by bulk weight, moisture content, and absence of foreign particulates. However, in this experiment, no physical parameter measured (bulk weight and 1,000 kernel weight) was significant at *P* ≤0.15 to predict DE or ME. The results obtained in the current study agreed with the data provided by Leeson [8] and Dale [9]. Such a result indicates that most of the observed variation in DE or ME between samples was the result of factors other than bulk weight and 1,000 kernel weight. Compared with the data of correlation coefficients (Table 5) among DE of DM values of samples in the study, EE, NDF, ash, GE and starch were 0.44, -0.32, -0.29, 0.25 and 0.38, respectively. Correlation coefficients and prediction equations with chemical composition clearly indicate that EE, starch and fibrous components predominantly determined the energy values. This observation is consistent with findings for barley by Fairbairn et al. [3], in which a prediction equation with an r^2^ of 0.89 for DE was reported if the similar variables were used.

Ether extract and starch were important prediction estimators of DE or ME in corn. The reasons may attribute that starch and dietary fat are important ingredients in diets because of its high energy value. Interestingly, the narrow range of GE levels in the 100 corn samples using herein (4,357 to 4,537 kcal/kg) did not seem to reduce the effectiveness of GE as an accurate estimator of DE or ME in corn. The reasons for this are unclear but the first estimate, based on a large set of experimental data, seems preferable. However, the coefficient of GE was positive with energy values in the study, and similar results were found in wheat (unpublished data) in our lab. Barley, wheat and sorghum showed the same result in Batterham’s study [35].

In agreement with most literature data [13,36,37], the presence of fiber in the diet of pigs reduces the ATTD of energy and nutrients. The content of NDF was another significant factor affecting the DE and ME variation in the present experiment. The prediction was improved when the different Van Soest fractions were included (Equations 3 in Table 6). This significant correlation between NDF and DE in other cereal grains has been observed by Perez et al. [38] and Fairbairn et al., [3]. Other researchers have found that the NDF content of corn was correlated with growing performance of pigs [6]. The reason may be that cellulose and lignin act as a diluent, thus lowering the energy content by displacing more digestible fractions such as starch. However, the dilution effect does not fully explain the changes in energy content. It may be that the presence of fiber can reduce the digestion of dietary nutrients. The results of the present study clearly show that ME content of corn can be accurately predicted from the DE content, because the correlation coefficient between DE and ME is approximately 0.95.

After testing the suitability of these models (Table 6) to predict the DE content of corn samples, our results showed that the difference between *vivo* method the prediction model was not significant (*P* > 0.16 in Table 8). This suggested that the accuracy of prediction models for DE was close to that obtained *in vivo*. In conclusion, the results of this study demonstrate that it is possible to estimate the DE and ME of corn from chemical variables. The best predictors for energy values were EE, starch, GE and NDF contents. Equation 4 (Table 6) and Equation 11 (Table 7) represent the best combination of accuracy and practicality when estimating DE and ME levels in corn.

## Abbreviations

DE: Digestible energy; ME: Metabolizable energy; CP: Crude protein; NDF: Neutral detergent fibre; ADF: Acid detergent fibre; ATTD: Apparent total tract digestibility; DDGS: Distillers dried grains with soluble; EE: Ether extract; GE: Gross energy; DM: Dry matter; RSD: Residual standard deviation.

## Competing interests

The authors declare that they have no competing interests.

## Authors’ contributions

QFL carried out the experiment trial, performed the statistics and drafted the manuscript. JJZ and XSP participated in design of the study. DWL participated animal trial. DFL and CHL conceived the study, and participated in its design and coordination. All authors read and approved the final manuscript.
